# Herbicidal properties of antimalarial drugs

**DOI:** 10.1038/srep45871

**Published:** 2017-03-31

**Authors:** Maxime G. Corral, Julie Leroux, Keith A. Stubbs, Joshua S. Mylne

**Affiliations:** 1 School of Molecular Sciences, The University of Western Australia, 35 Stirling Highway, Crawley, Perth 6009, Australia; 2 The ARC Centre of Excellence in Plant Energy Biology, 35 Stirling Highway, Crawley, Perth 6009, Australia

## Abstract

The evolutionary relationship between plants and the malarial parasite *Plasmodium falciparum* is well established and underscored by the *P. falciparum* apicoplast, an essential chloroplast-like organelle. As a result of this relationship, studies have demonstrated that herbicides active against plants are also active against *P. falciparum* and thus could act as antimalarial drug leads. Here we show the converse is also true; many antimalarial compounds developed for human use are highly herbicidal. We found that human antimalarial drugs (e.g. sulfadiazine, sulfadoxine, pyrimethamine, cycloguanil) were lethal to the model plant *Arabidopsis thaliana* at similar concentrations to market herbicides glufosinate and glyphosate. Furthermore, the physicochemical properties of these herbicidal antimalarial compounds were similar to commercially used herbicides. The implications of this finding that many antimalarial compounds are herbicidal proffers two novel applications: (i) using the genetically tractable *A. thaliana* to reveal mode-of-action for understudied antimalarial drugs, and (ii) co-opting antimalarial compounds as a new source for much needed herbicide lead molecules.

Malaria is caused by protozoan endoparasites that belong to the genus *Plasmodium* and the phylum Apicomplexa with transmission between human hosts performed by female *Anopheles* mosquitoes. Interestingly, these protozoan parasites have much in common with plants^1^. The most obvious plant connection is that most apicomplexan parasites harbour a plastid similar to the chloroplast of plants and algae, known as the apicoplast. The origin of the apicoplast in *Plasmodium* and other apicomplexans is thought to be from secondary endosymbiosis whereby a heterotrophic eukaryote engulfed a red algal ancestor, the latter being retained as a plastid^2^,^3^,^4^,^5^. The apicoplast is non-photosynthetic, but critical for the survival of *Plasmodium* as it is involved in the synthesis of fatty acids, iron-sulphur clusters and haem, that are essential for the liver and mosquito stages of the parasite life cycle^4^,^5^,^6^,^7^. The production of isoprenoid precursors by the apicoplast is essential for the human blood stage^8^,^9^. In addition to this prominent chloroplast relic, many genes thought to have originated from the red algal ancestor have also transferred into the nuclear genome of apicomplexan parasites in a similar manner to chloroplast-encoded genes in plants^10^. Of note is that comparison of the *P. falciparum* genome with other annotated eukaryotic genomes revealed greater similarity of *P. falciparum* to the flowering plant *Arabidopsis thaliana* than any other non-apicomplexan taxa^1^.

*A. thaliana* was the first plant to have its genome sequenced^11^ and has become one of the most heavily studied model organisms in genetics and molecular biology. It is genetically tractable, has a small genome (135 MB, 5 chromosomes), a short life cycle and is easy to grow. A wealth of natural variants and gene knock-out lines are available as well as classical point mutants induced by mutagens such as ethyl methanesulfonate^12^.

The evolutionary relationship between apicomplexans and plants is not only apparent at the organelle level, but also from the activity of herbicides against *Plasmodium*. Indeed, pharmaceutical and agrochemical leads often show similarities in structure and activity^13^. Delaney *et al*.^14^ showed that the 20 most frequently used side chain groups in pharmaceutical drugs are found with similar frequency in herbicides. Coupled with this is that the target sites or biological processes that human drugs and agrochemicals interact with are sometimes shared. As such, compounds toxic to plants might similarly affect other organisms like *Plasmodium* species with examples available for commercial herbicides^13^. The widely used commercial herbicide glyphosate inhibits the *P. falciparum* asexual blood-stage *in vitro*, suggesting a shikimate pathway is shared with plants^15^. The commercial herbicide endothall inhibited growth of a chloroquine-sensitive *P. falciparum* strain with an IC_50_ of ≈8 μM^16^. Dinitroaniline-type herbicides also have activity against protozoal parasites including *Plasmodium* species^17^,^18^,^19^. In a larger study, the activity of 680 commercial agrochemicals including insecticides, fungicides and herbicides were systematically tested *in vitro* and *in vivo* on *Plasmodium* species, *Leishmania* species and trypanosomal parasites. Some of the compounds were highly active against protozoan pathogens and have potential as drug leads^20^.

Along with commercial herbicides, relationships between herbicidal and antimalarial compounds at the pre-development stage have also been reported in the literature. Witschel *et al*.^21^ investigated inhibitors of plant serine hydroxymethyltransferase, a key enzyme in the folate cycle, and showed many had significant activity against both *P. falciparum* and *P. vivax* enzymes. The antibiotic fosmidomycin which targets 1-deoxy-D-xylulose-5-phosphate reductoisomerase has been shown to be antimalarial^22^. Finally, the aryl bis-sulfonamides which are inhibitors of 2-methylerythritol 2,4-cyclodiphosphate synthase, were found to inhibit both *A. thaliana* and *P. falciparum* enzymes *in vitro*^23^.

Despite there being considerable interest in using herbicides as antimalarial compounds, applications in the reverse direction are lacking. Thus we explored this rationale, namely whether compounds specifically designed to kill *Plasmodium* and other apicomplexans are also active against plants. We found many antimalarial drugs to be herbicidal, which offers two new avenues for future research; the first and most obvious is to use the significant knowledge of antimalarial drugs to consider new chemistries or modes-of-action for herbicides. The second and more radical possibility is to use the genetically tractable *A. thaliana* to explore what could be shared modes-of-action for understudied antimalarial drugs.

## Results

### The effect of herbicides on *A. thaliana* growth

**Figure 1:**
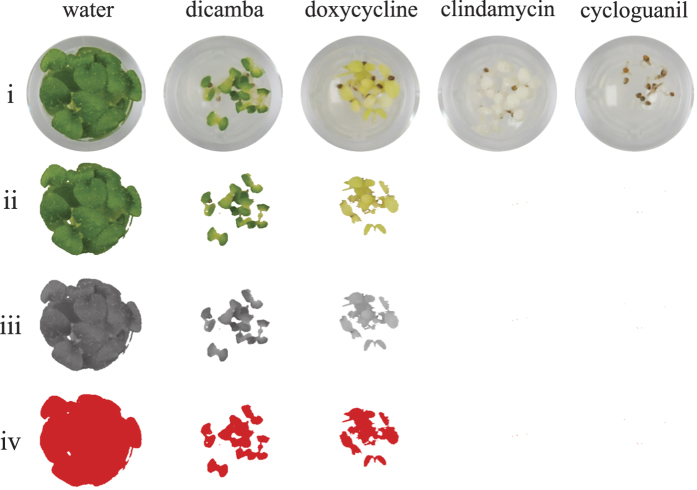
Quantification of plant growth. To quantify growth within a plate using ImageJ, an original image (**i**) was filtered using the ‘Threshold Colour’ plug-in so that only the shades of green are retained (**ii**) before the image was converted to an 8-bit image (**iii**) and threshold adjusted to convert grey shades into red pixels that are measurable by ImageJ (**iv**). In this way, from an image, the green pixels can be counted and a value for total area in mm^2^ obtained.

**Figure 2:**
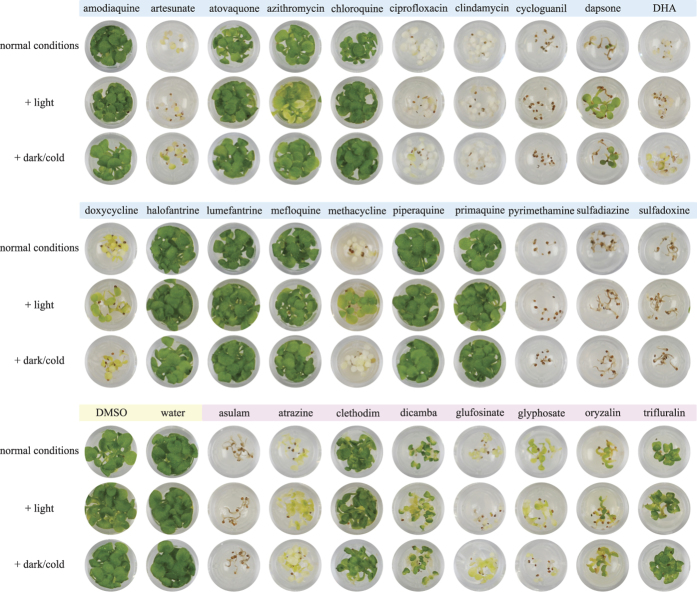
Growth of *A. thaliana* on media containing herbicides and antimalarials. *A. thaliana* was raised from seeds on growth media containing 20 μg/mL of antimalarials and antibiotics (top row, middle row) in comparison to negative controls (DMSO, water) and known herbicides (bottom row). To test light and solution instability, media was pre-treated at 22 °C with light for 1 week (+light) or left at 4 °C in the dark for 1 week (+dark/cold) before sowing seeds. For each compound and condition, three replicates were done and a representative image is shown.

To test the effect of compounds, sterile *A. thaliana* seeds were sown on media containing sugar, salts and vitamins, and supplemented with a compound of interest. After two weeks, the extent of growth inhibition for each compound was assessed by analysing images with ImageJ (Fig. 1). Most of the herbicides chosen (Table 1) inhibited *A. thaliana* germination and growth at 20 μg/mL (Fig. 2). Asulam inhibited growth after radicle emergence, whereas atrazine, glufosinate and glyphosate halted growth after expansion of what were chlorotic cotyledons. Atrazine was the only herbicide tested that displayed apparent instability, being less effective after one week of light (Supplementary Fig. 1). The concentration 20 μg/mL was sub-lethal for oryzalin and dicamba which across conditions inhibited growth by 76% and 64% respectively with arrested growth at the cotyledon stage. Trifluralin was less effective at 20 μg/mL, inhibiting growth by ≈50%. This is probably due to the well-known instability under sunlight and volatility of dinitroanilines^24^,^25^. Clethodim is a monocot-specific herbicide and inhibited *A. thaliana* (dicot) growth only by 3–30% and allowed true leaves to emerge (Supplementary Fig. 1). Overall these control compounds highlight that even successful market herbicides range in efficacy against *A. thaliana* and similarly, an antimalarial drug might not affect *A. thaliana*, but could remain highly effective against other, non-tested plant species.

### Antimalarial compounds with herbicidal activity

Of the twenty antimalarial compounds tested, (Table 1) eleven were active against *A. thaliana* at 20 μg/mL (Fig. 2). The artemisinin-based drugs artesunate and dihydroartemisinin (DHA) showed 98% growth inhibition and induced chlorosis in the cotyledons. Ciprofloxacin, clindamycin and methacycline allowed germination and full expansion of cotyledons that were completely white. Methacycline efficacy was reduced by light pre-treatment, but not dark pre-treatment, suggesting light sensitivity. Doxycycline, a tetracycline like methacycline, also induced chlorosis, but the plants yellowed rather than fully bleaching. Doxycycline showed some light sensitivity, but less than methacycline. The antifolates pyrimethamine, cycloguanil, sulfadoxine and sulfadiazine inhibited development allowing only emergence of the radicle. Dapsone, another antifolate, was effective under normal conditions, but was unstable showing reduced efficacy after light or dark pre-treatment of the medium, although the latter was not statistically significant at 95% confidence (*P*_*1*_ = 0.056). Amodiaquine, atovaquone, azithromycin, chloroquine, halofantrine, lumefantrine, mefloquine, piperaquine and primaquine did not have any herbicidal activity against *A. thaliana* at 20 μg/mL (Supplementary Fig. 1).

### Calculating LD_50_ for herbicidal antimalarials

**Figure 3:**
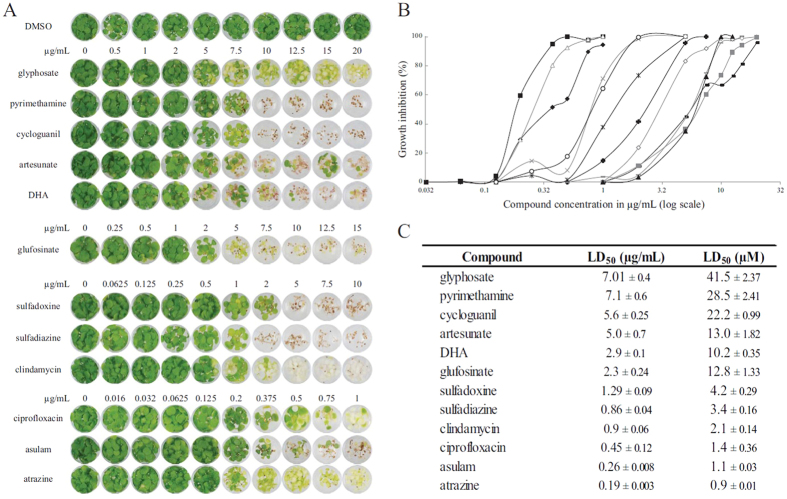
Potency of herbicidal antimalarials. (**A**) *A. thaliana* seeds raised on a concentration gradient of herbicides, antimalarials, and antibiotics. (**B**) Dose-response curves for all compounds, except DMSO. The X-axis is a log scale with concentrations tested. The Y-axis is given as percentage growth inhibition compared to DMSO control. Each data point is a mean value from six replicates (details in Supplementary Table 2). (◆) Ciprofloxacin, (■) atrazine, (△) asulam, (×) sulfadiazine, (×) sulfadoxine, (◯) clindamycin, (✚) glufosinate ammonium, (▬) artesunate, (▭) cycloguani, (◇) DHA, (□) glyphosate, and (▲) pyrimethamine. (**C**) LD_50_ (μg/mL) measured for all compounds.

The antimalarial compounds that were stable and herbicidal at 20 μg/mL, were tested at a range of concentrations to determine potency alongside the herbicides glyphosate, glufosinate, asulam and atrazine as controls (Fig. 3A). Dose-response curves demonstrated a range of potencies (Fig. 3B,C) with the herbicides atrazine and asulam being the most potent compounds tested, with LD_50_ values of 0.19 μg/mL and 0.26 μg/mL, respectively. Of the eight antimalarial and antibiotic compounds tested here, the antibiotics ciprofloxacin (LD_50_ = 0.45 μg/mL) and clindamycin (LD_50_ = 0.9 μg/mL), and the antifolates sulfadiazine (LD_50_ = 0.86 μg/mL) and sulfadoxine (LD_50_ = 1.29 μg/mL) were the most herbicidal, notably with greater potency than glufosinate and glyphosate. The artemisinin derivate DHA was slightly more herbicidal than artesunate with LD_50_ values of 2.9 μg/mL and 5.0 μg/mL respectively. The antifolate cycloguanil (LD_50_ = 5.6 μg/mL) was of similar potency to pyrimethamine (LD_50_ = 7.1 μg/mL) and glyphosate (LD_50_ = 7.0 μg/mL). These data show some antimalarial compounds have similar or greater potency than the active ingredients of market herbicides.

### The physicochemical properties of antimalarials *vs* herbicides

**Figure 4:**
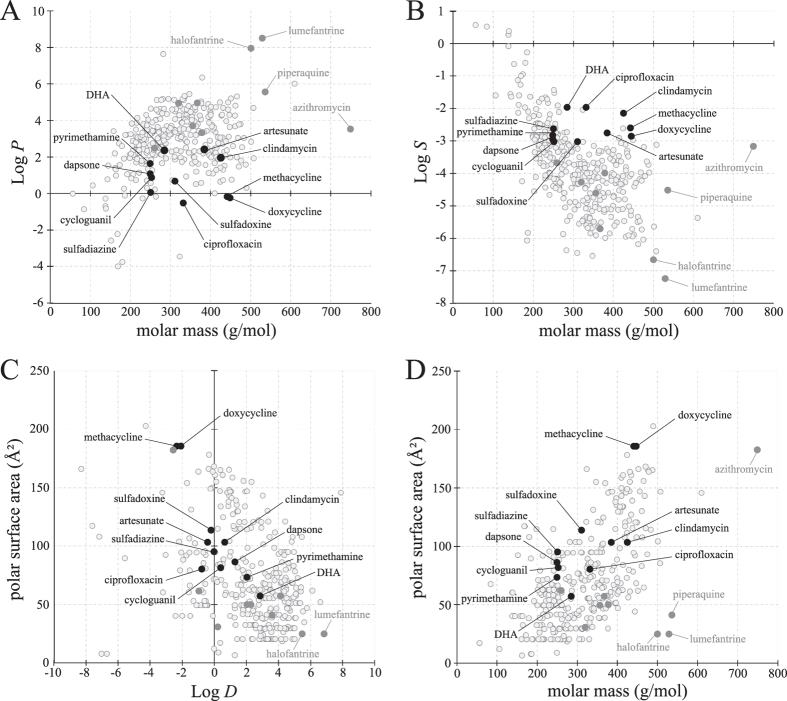
Physicochemical properties of antimalarial compounds *versus* herbicides. Representative charts (**A**–**C**) were extracted using an interactive database containing the physicochemical properties of 334 commercial herbicides^26^. New data points (i.e. compounds studied here) were added to the database and plotted on graphs comparing two chemical properties, (**A**) molar mass *vs* lipophilicity (Log *P*), (**B**) molar mass *vs* aqueous solubility (Log *S*), (**C**) distribution coefficient (Log *D) vs* polar surface area (Å^2^) and (**D**) molar mass *vs* polar surface area (Å^2^). Cluster analyses show the general trends into which the antimalarial compounds fall. Black dots represent the antimalarial compounds that significantly inhibited plant growth, whereas solid grey dots represent antimalarial compounds with poor or no herbicidal activity. Transparent dots represent the 334 herbicidal compounds of the original database^26^.

Although the physicochemical properties of herbicides and pharmaceuticals have much in common^26^, there are some consistent differences. Notably this includes the number of hydrogen bond donors, which in herbicides is rarely greater than one, whereas oral drugs can have up to five^27^. To determine whether the physicochemical properties of a specific antimalarial explains its herbicidal efficacy we subjected the tested compounds to a cluster analysis (Fig. 4) using an interactive database that compares and displays properties of 334 commercial herbicides^26^. In general, antimalarials have similar physicochemical properties to herbicides however compounds without herbicidal activity were often at the fringes, or outliers, when their physicochemical properties were mapped onto those of herbicides (Fig. 4). For example the larger compounds halofantrine, lumefantrine and piperaquine were outliers when polar surface area was plotted against molecular mass (Fig. 4D). Although their polar surface area is similar to herbicides with molecular masses of 150–400, compared to herbicides with molecular mass above 500, the polar surface area of these three compounds is exceptionally low. Methacycline and doxycycline, which were herbicidal but light sensitive, were outliers in graphs of polar surface area *versus* distribution coefficient (Fig. 4C) and polar surface area *versus* molar mass (Fig. 4D). In general though physicochemical properties alone could not explain the efficacy for some compounds. Another explanation is that some antimalarial modes-of-action will target processes that do not exist in plants (e.g. haem-binding by chloroquine).

## Discussion

The present study aimed to assess whether antimalarial compounds that have been historically used to treat malaria are also active as herbicides against plants. We tested twenty compounds ranging from highly specific antimalarial drugs to antimalarial antibiotics and of these, eleven were as potent or more potent than some commercial herbicides (Fig. 2, Supplementary Fig. 1). As plants and malarial parasites share a common evolutionary history, we believe that inhibitors of malarial cellular components might also be active against the orthologous components in plants. Such compounds could offer novel chemistries with new modes-of-action for the development of plant-specific inhibitors. Herbicide discovery has dramatically declined since the widely used herbicide glyphosate and glyphosate-resistant crops were implemented^28^. Just over twenty herbicide modes-of-action are known and no new mode-of-action has been used for herbicides released in the past two decades^29^. The importance of designing novel herbicidal chemistries is further underlined when looking at the alarming number of 252 weed species that have evolved resistance to one or more herbicidal mode-of-action. To glyphosate alone there are 269 reported cases of resistance in 27 countries from all over the globe^30^,^31^.

Inhibitors active against malarial parasites but showing only mild or no effect on plants can be explained by; (i) the active compound has no molecular targets *in planta*, (ii) the compound has a molecular target *in planta*, but interaction is prevented by physical barriers (e.g. root epidermis, leaf cuticle, lack of membrane transporters), (iii) the inhibitor has a target but is rapidly catabolised by the plant cell to be inactive.

The 4-aminoquinoline drugs chloroquine and amodiaquine have been widely used to prevent and treat malaria until resistant *P. falciparum* emerged^32^,^33^. In *Arabidopsis*, neither compound was herbicidal. The mechanism of action of chloroquine is well established, and the chemically similar amodiaquine is assumed to act in a similar fashion^34^. During haemoglobin catabolism, free haem is converted by *Plasmodium* into its insoluble and non-toxic form called haemozoin. Chloroquine binds to free haem, which prevents its conversion and the parasite becomes susceptible to haem toxicity^35^,^36^. Resistance to chloroquine is linked to mutations in membrane proteins of the parasite digestive vacuole^37^,^38^. Although the mode-of-action of mefloquine, halofantrine, piperaquine and lumefantrine remains unclear, evidence suggests that they also interfere with haemozoin formation^39^. As haemoglobin and the process of converting haem to haemozoin is absent in plants, this may explain the lack of herbicidal activity observed for all these compounds.

Primaquine is an important drug active in the liver stage of *Plasmodium* infections, mainly in *P. vivax*^40^. Its mechanism of action, although unclear, is thought to involve disruption of mitochondrial function^41^. It is suggested that primaquine metabolism in the body leads to the production of reactive intermediates and free radicals which account for its activity^42^. As primaquine showed no herbicidal activity against *Arabidopsis* and given that primaquine metabolism is required for its antimalarial activity, it is possible that primaquine is not metabolised *in planta*, thus remains inactive.

The macrolide antibiotic azithromycin is a semi-synthetic erythromycin derivative. Although it has activity against *P. falciparum*, especially in synergy with quinine^43^, azithromycin is a relatively weak antimalarial both in mono- and combination therapy^44^. In bacteria, azithromycin binds to the 50S ribosomal subunit and blocks transpeptidation and polypeptide translocation during translation, therefore inhibiting protein synthesis^45^. *In vitro* studies using *Plasmodium* species also showed that azithromycin could inhibit parasite invasion of red blood cells, a role independent of inhibition of protein synthesis^46^. In our study, azithromycin did not display herbicidal activity, possibly due to a lack of target. Alternatively, azithromycin might be able to target ribosomal functions in *Arabidopsis* as it does in bacteria, but lacks the required physicochemical properties to be active in plants (Fig. 4A,B and D).

Doxycycline and methacycline are broad-spectrum tetracycline antibiotics. In prokaryotes, tetracyclines block protein translation by binding to the ribosomal 30S subunit^47^. Although the mechanism of action for tetracyclines in *Plasmodium* remains unclear, doxycycline has been shown to act by disrupting the expression of apicoplast-encoded genes^48^. In *Arabidopsis*, doxycycline and methacycline induced bleaching of the cotyledons (Fig. 2). This might indicate that tetracyclines affect chloroplast function or have downstream effects on protein synthesis that leads to chlorophyll depletion. Although these compounds showed potential as herbicide leads, both compounds are light-sensitive, a downside of tetracycline antibiotics^49^. For obvious reasons, photo-sensitivity limits a compound being considered as an herbicidal lead.

Clindamycin is a semi-synthetic lincomycin derivative with antibiotic, antimalarial and anti-apicomplexan (e.g. *Toxoplasma gondii*) activities^50^. In prokaryotes, clindamycin binds the 50S ribosomal subunit and prevents interaction of peptidyl-tRNA with the ribosome, thereby inhibiting protein translation^51^. In protozoan parasites, clindamycin targets the apicoplast large rRNA subunit as inferred from clindamycin-resistant *T. gondii* mutants that harbour a point mutation in the apicoplast large rRNA subunit^52^. Clindamycin was revealed to be one of the most herbicidal compounds, with greater activity than glufosinate and glyphosate. Except for azithromycin, all inhibitors of protein synthesis tested here were herbicidal. Their mode-of-action in *Arabidopsis* could be similar to that in bacteria and protozoa. In terms of potency, ciprofloxacin, a quinolone-based antibiotic, was also more active than glufosinate and glyphosate in the plate assay (Fig. 4). It was recently shown that *A. thaliana* DNA gyrase, a type II topoisomerase, is the target of ciprofloxacin through the identification of a point mutation in the *ATGYRA* gene responsible for ciprofloxacin resistance in an *A. thaliana* genetic mutant^53^. Of the ∼20 known herbicide modes-of-action, DNA gyrase is not a current target of any market herbicide^26^, making the mode-of-action of ciprofloxacin an attractive avenue for the development of new herbicides.

Artesunate and dihydroartemisinin (DHA) are semi-synthetic derivatives of the antimalarial agent artemisinin, a natural product found in the Chinese herb *Artemisia annua*^54^. Artemisinin derivatives are the most widely used antimalarial drugs and are important components of artemisinin derivative-based combination therapies for the treatment of uncomplicated malaria^55^. The molecular mechanisms by which artemisinins act as antimalarials are still unclear and multiple modes-of-action have been proposed including interfering with iron-haem interactions and stimulating the formation of radical intermediates^56^,^57^, targeting of a calcium-ATPase transport protein, *Pf*ATP6 which affects calcium levels required for parasite invasion in red blood cells^58^ and interference in the formation of polyphosphorylated phosphoinositides, which are important regulators of signalling and trafficking functions in most eukaryotes^59^. Both artesunate and DHA were potent herbicides and although using such important compounds in an herbicidal context is unlikely, using *A. thaliana* as a model to gain insight into its mode-of-action might be sought.

The antifolates are important inhibitors of folate synthesis and metabolism. Folate and its derivatives are essential co-factors for DNA synthesis and production of several amino acids^60^. Of the enzymes involved in the folate pathway, dihydrofolate reductase (DHFR) and dihydropteroate synthase (DHPS) are well described as the molecular targets of important antifolate drugs. In both *Plasmodium* parasites and plants, DHFR is part of a bifunctional protein with thymidylate synthetase (DHFR-TS). Plant DHPS is also part of a bifunctional protein with hydroxymethylpterin pyrophoskinase (PPPK-DHPS)^61^. Pyrimethamine and cycloguanil inhibit *P. falciparum* DHFR-TS by binding DHFR^62^, whereas the sulfa-based drugs sulfadoxine, sulfadiazine and dapsone act by binding DHPS in bacteria and protozoans^63^,^64^. Here, all DHFR and DHPS inhibitors tested were herbicidal with dapsone the only compound that displayed photosensitivity. In addition all the compounds display good herbicidal physicochemical properties and as *Arabidopsis* and *Plasmodium* share the bifunctional-type DHFR and DHPS proteins, we expect the antifolate drugs tested here to have the same mode-of-action in plants and could present interesting starting points for new herbicidal development.

The mode-of-action for many antimalarials, some in use for decades, are unclear or unknown. As a result this prevents a greater understanding of the biological action of the compound and also prevents development of more potent compounds for the target. Some examples are the artemisinin-based compounds whose mode-of-action remains controversial and other, novel and promising antimalarial compounds whose modes-of-action remain to be investigated^65^. If the evolutionary relationship between *Arabidopsis* and *Plasmodium* is close enough that the antimalarial compounds have the same mode-of-action and same targets, the genetically tractable *A. thaliana* might have a place to study the biological action of these materials. Chemically mutagenized seeds could be sown and grown under drug selection to find resistant plants and map-based cloning of the resistance lesions could reveal the target or explain the compounds mode-of-action. Such a strategy has been employed by Jander *et al*.^66^ in the study of herbicide resistance where screens that used chlorsulfuron and imazethapyr found lesions in their target, acetolactate synthase, which is crucial for the biosynthesis of the branched-chain amino acids. Fluoroquinolones such as ciprofloxacin, are known topoisomerase inhibitors and have been shown to have potential as antimalarial compounds^67^. A mutagenesis strategy using *A. thaliana* seeds and screening for resistant plants found a genetic mutation in the equivalent plant target of ciprofloxacin, DNA gyrase A^53^ thus demonstrating the tractability of using *A. thaliana* to identify putative modes-of-action. Such a forward genetic approach using *A. thaliana* would not work for all antimalarial drugs, but would be very useful for protein targets where gene loss or single amino acid substitutions can render plants resistant to the compound.

## Conclusions

Our study shows that compounds designed for treating malaria also display herbicidal activity. As antimalarial drugs are non-toxic to humans and display, in general, good physicochemical properties (e.g. water solubility) these make them attractive bioactive molecules. Due to the alarming recent increases in herbicide resistance, such compounds could offer a good starting point in the design of new herbicidal chemistries. Here we show that some antimalarials are as potent, or more potent, than the active components of commercial herbicides. From these observations, a few questions are raised; do all herbicidal antimalarials act in plants in similar ways as in malaria parasites, that is, do they inhibit similar proteins or affect the same pathways? If so, mutagenesis of the genetically tractable *A. thaliana* and screening for drug resistant plants could reveal the modes-of-action for understudied antimalarial drugs in the same way that forward genetics can reveal targets for herbicides. Another use of this cross-over concept is whether the significant resources that have become available for the development of antimalarial lead compounds could also be used to develop novel herbicides. The alarming rise globally in herbicide resistance in weeds^30^ is driving the agrochemical industry to search for new lead compounds and ideally, compounds with novel modes-of-action.

## Methods

### Germination assays

*Arabidopsis thaliana* ecotype Col-0 seeds were surface sterilised for 3 min in 70% ethanol, followed by 1 min in 100% ethanol and then soaked for 3 min in sterile water. Seeds were re-suspended in sterile 0.1% agar and incubated at 4 °C for 3 days to synchronise subsequent germination. Following this stratification treatment, seeds were sown on 96-well microplates (TrueLine, USA) with each well containing 0.25 mL of growth medium (1% agar, 1% glucose, 0.45% Murashige & Skoog salts with vitamins, 0.3% 2-(*N*-morpholino)-ethanesulfonic acid (MES) (v/v), pH 5.7) and the appropriate dose of DMSO, antimalarial, antibiotic or herbicide. Plates were sealed with porous tape and transferred to a chamber at 22 °C for two weeks under 16 h days and 60% relative humidity before a photo of each plate was taken. To initially determine the herbicidal nature of the antimalarial compounds, a screen using a single concentration was performed (20 μg/mL). If herbicidal activity was displayed, the assay was repeated with a range of concentrations. To investigate compound stability, plates were pre-treated for one week prior to seed sowing either by being left under lights at 22 °C or in the dark at 4 °C. Growth of seeds sown on these pre-treated plates were compared to seeds sown on freshly prepared plates.

### Commercial herbicides, antimalarials and antibiotics

Twenty-eight compounds were tested for an herbicidal effect on *A. thaliana*. Eight commercially available herbicides were compared to twenty antimalarial compounds (Table 1). Amodiaquine, chloroquine, ciprofloxacin, glufosinate ammonium, glyphosate and oryzalin were obtained from Sigma-Aldrich. Artesunate was obtained from Pharbaco Central Pharmaceutical J.S.C., mefloquine from Roche, and asulam from Sapphire Bioscience. The rest (atovaquone, atrazine, azithromycin, clethodim, clindamycin, cycloguanil, dapsone, dicamba, dihydroartemisinin, doxycycline, halofantrine, lumefantrine, methacycline, piperaquine, primaquine, pyrimethamine, sulfadiazine, sulfadoxine, trifluralin) were sourced from AK Scientific. Stock solutions were prepared in DMSO, except chloroquine and glyphosate, which were prepared in water. Initial screening was at 20 μg/mL and the compounds that showed herbicidal activity were tested at a range of concentrations with DMSO as the negative control and four herbicides as positive controls.

### Quantification of growth inhibition

To quantify growth inhibition on plates, photos were analysed using ImageJ (National Institutes of Health, 1.47 v). The ‘Threshold Colour’ plug-in was adjusted to specifically follow green pixels as a correlation of healthy seedlings, as opposed to chlorotic leaves which display pale yellow or white pixel characteristics. To display only green channels the settings ‘Hue’ was 50–110, ‘Saturation’ was 125–255 and ‘Brightness’ 30–255. Images were then converted into 8-bit format and the threshold adjusted to change the greyscale into red pixels that could be measured (ImageJ). Total green pixels were then compared to total well area (mm^2^) and data were normalised against the negative control (DMSO or water) to provide percentage inhibition.

### Physicochemical properties of herbicidal compounds

The physicochemical properties of the tested compounds were calculated as described in Gandy *et al*.^26^ and displayed in the context of the physicochemical properties for 334 known herbicides.

## Additional Information

**How to cite this article**: Corral, M. G. *et al*. Herbicidal properties of antimalarial drugs. *Sci. Rep.* **7**, 45871; doi: 10.1038/srep45871 (2017).

**Publisher's note:** Springer Nature remains neutral with regard to jurisdictional claims in published maps and institutional affiliations.

## Supplementary Material

Supplementary Information

## Figures and Tables

**Table 1:** Compounds tested in this study.

Herbicides	CAS #
asulam	3337-71-1
atrazine	1912-24-9
clethodim	99129-21-2
dicamba	1918-00-9
glufosinate ammonium	77182-82-2
glyphosate	1071-83-6
oryzalin	19044-88-3
trifluralin	1582-09-8
**Antimalarial**	
amodiaquine	6398-98-7
artesunate	88495-63-0
atovaquone	95233-18-4
azithromycin	83905-01-5
chloroquine	50-63-5
ciprofloxacin	85721-33-1
clindamycin	58207-19-5
cycloguanil	152-53-4
dapsone	80-08-0
dihydroartemisinin	71939-50-9
doxycycline	10592-13-9
halofantrine	69756-53-2
lumefantrine	82186-77-4
mefloquine	53230-10-7
methacycline	3963-95-9
piperaquine	4085-31-8
primaquine	90-34-6
pyrimethamine	58-14-0
sulfadiazine	68-35-9
sulfadoxine	2447-57-6
