# Supplementary material for: Herbicidal properties of antimalarial drugs
Source: Sci Rep. 2017 Mar 31;7:45871. doi: 10.1038/srep45871 (PMC5374466; doi:10.1038/srep45871)
Supplement: Supplementary Information [file srep45871-s1.pdf]

Supplementary Information:

Herbicidal properties of antimalarial drugs

Maxime G. Corral, Julie Leroux, Keith A. Stubbs, Joshua S. Mylne

Supplementary Figures

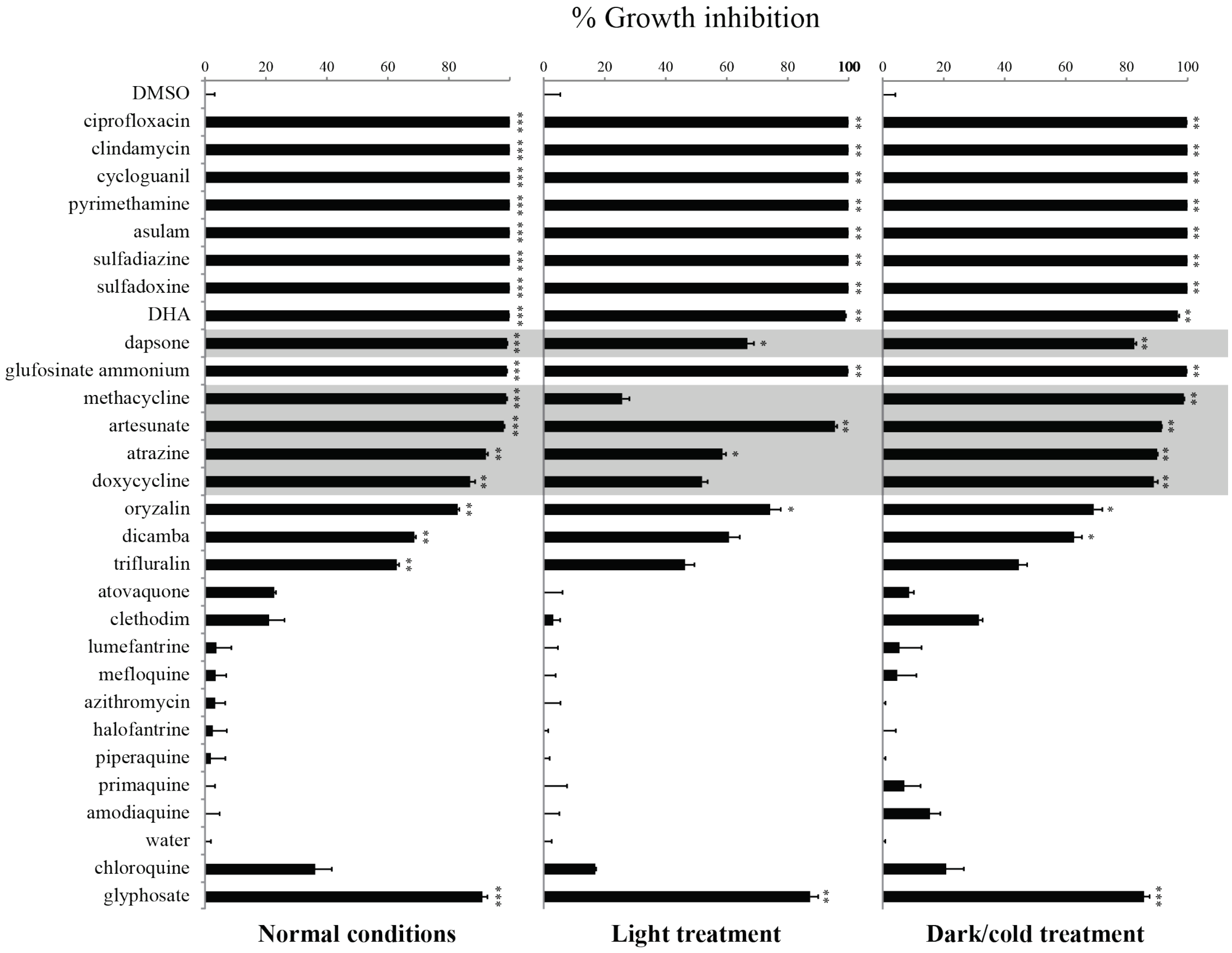

Supplementary Figure 1 | *A. thaliana* growth inhibition by herbicides and antimalarial compounds

The effect of herbicides, antimalarials and antibiotics is given as a percentage of growth inhibition under normal conditions, light treatment and dark/cold treatment. DMSO and water were used as controls. Values are given as the mean of % growth inhibition from three replicates and the error bars represent standard error. Statistical differences from the DMSO control were determined using a Student's *t*-test and are represented with an asterisk (*$P < 0.5$, **$P < 0.1$ and ***$P < 0.01$). Grey shading highlights the compounds for which significant differences were found between treatments.

Supplementary Tables

Supplementary Table 1 | *A. thaliana* growth inhibition by herbicides and antimalarial compounds

Values are given as the mean (± SE) of the total area (mm^2) of seedlings grown in a single well (using method illustrated in **Fig. 1**) from three replicates. Data was normalised against data from the relevant control (DMSO or water). Asterisks represent significant differences between a compound and its control according to Student's *t*-test at $P < 0.05$ (*), $P < 0.01$ (**) and $P < 0.001$ (***). Superscript letters indicate significant differences between conditions for the same compound. In this instance, when *P* values are mentioned, P_1 refers to as values compared between 'normal conditions' and 'light treatment', P_2 refers to as values compared between 'light treatment' and 'dark/cold treatment', and P_3 refers to as values compared between 'normal conditions' and 'dark/cold treatment'.

	Normal conditions	Light treatment	Dark/Cold treatment
**DMSO**	30.03 ± 3.23	26.72 ± 5.42	29.40 ± 4.19
amodiaquine	30.22 ± 4.81	34.84 ± 5.17	24.85 ± 3.40
primaquine	30.15 ± 3.30	32.67 ± 7.70	27.32 ± 5.36
piperaquine	29.46 ± 4.81	36.86 ± 1.97	34.54 ± 0.93
halofantrine	29.25 ± 4.59	39.46 ± 1.49	34.88 ± 4.31
azithromycin	29.02 ± 3.29	27.04 ± 5.52	32.18 ± 0.94
mefloquine	28.97 ± 3.44	32.06 ± 3.95	27.99 ± 6.29
lumefantrine	28.90 ± 4.88	34.71 ± 4.68	27.78 ± 7.22
clethodim	23.71 ± 5.04	25.88 ± 2.25	20.12 ± 1.20
atovaquone	23.21 ± 0.55	28.20 ± 6.19	26.86 ± 1.58
trifluralin	11.13** ± 0.78	14.33 ± 3.06	16.26 ± 2.65
dicamba	9.39** ± 0.49	10.47 ± 3.53	10.93* ± 2.53
oryzalin	5.13** ± 0.55	6.88* ± 3.48	9.05* ± 2.81
doxycycline	3.93** ± 1.66 [a,c]	12.84 ± 1.82 [b]	3.26** ± 1.33 [a,c]
atrazine	2.37** ± 0.69 [a,c]	11.06* ± 1.19 [b]	2.94** ± 0.24 [a,c]
artesunate	0.61*** ± 0.29 [a]	1.20** ± 0.70 [a,b]	2.50** ± 0.04 b
methacycline	0.37*** ± 0.29 [a,c]	19.85 ± 2.42 [b]	0.37** ± 0.20 [a,c]
glufosinate	0.30*** ± 0.08	0.05** ± 0.02	0.07** ± 0.05
dapsone	0.29*** ± 0.15 [a]	8.87* ± 2.14 [a,b]	5.14** ± 0.64 [b]
DHA	0.04*** ± 0.04	0.28** ± 0.18	0.94** ± 0.50
sulfadoxine	0***	0**	0**
sulfadiazine	0***	0**	0**
asulam	0***	0**	0**
cycloguanil	0***	0**	0**
pyrimethamine	0***	0**	0**
clindamycin	0***	0**	0**
ciprofloxacin	0***	0**	0.06** ± 0.06
**Water**	33.74 ± 1.97	37.03 ± 2.64	37.75 ± 0.88
chloroquine	21.54 ± 5.46	30.75 ± 0.24	29.88 ± 5.76
glyphosate	3.04*** ± 1.65	4.68** ± 2.63	5.39*** ± 1.81

Supplementary Table 2 | Potency of herbicidal antimalarial compounds versus controls

Values are given as the mean (± SE) of the total area (mm^2) of seedlings grown in a single well (using method in **Fig. 1**) from six replicates. Compounds were tested at different concentrations and data were plotted as dose-response curves (**Fig. 3B**).

Concentration (µg/mL)	asulam	atrazine	ciprofloxacin	clindamycin	sulfadiazine	sulfadoxine	glufosinate	DHA	artesunate	cycloguanil	pyrimethamine	glyphosate	DMSO
**0**	39.99 ± 1.0	41.11 ± 0.7	41.67 ± 0.9	42.71 ± 1.1	38.01 ± 1.5	40.36 ± 0.9	37.72 ± 0.8	38.98 ± 1.5	40.93 ± 1.5	39.65 ± 1.1	38.99 ± 0.9	42.33 ± 1.5	39.40 ± 1.2
**0.004**	39.23 ± 0.7	40.82 ± 0.5	41.07 ± 1.4	-	-	-	-	-	-	-	-	-	-
**0.008**	38.32 ± 1.7	40.3 ± 1.1	38.89 ± 1.4	-	-	-	-	-	-	-	-	-	-
**0.016**	35.93 ± 1.8	39.29 ± 0.8	38.06 ± 1.4	-	-	-	-	-	-	-	-	-	-
**0.032**	39.51 ± 1.6	41.47 ± 0.9	38.63 ± 1.1	-	-	-	-	-	-	-	-	-	-
**0.0625**	40.53 ± 1.4	41.27 ± 0.6	39.38 ± 0.6	44.23 ± 1.4	32.39 ± 2.5	39.98 ± 1.0	-	-	-	-	-	-	-
**0.125**	41.6 ± 0.6	39.69 ± 0.4	38.7 ± 0.8	44.38 ± 1.0	33.64 ± 2.4	42.22 ± 1.2	-	-	-	-	-	-	-
**0.2**	29.52 ± 2.5	16.84 ± 3.6	27.88 ± 2.2	-	-	-	-	-	-	-	-	-	-
**0.25**	-	-	-	41.08 ± 1.0	28.77 ± 3.5	40.36 ± 1.5	36.80 ± 1.6	-	-	-	-	-	-
**0.375**	8.25 ± 1.3	2.17 ± 0.8	19.14 ± 3.4	-	-	-	-	-	-	-	-	-	-
**0.5**	3.2 ± 1.0	0.06 ± 0.02	16.85 ± 1.8	30.91 ± 2.3	41.57 ± 1.4	36.53 ± 1.4	38.68 ± 0.8	37.98 ± 1.1	42.68 ± 1.5	41.65 ± 1.4	40.03 ± 0.3	41.71 ± 1.5	39.76 ± 1.7
**0.75**	0.83 ± 0.6	0.97 ± 0.5	4.15 ± 1.2	-	-	-	-	-	-	-	-	-	-
**1**	0	0	2.24 ± 1.4	9.73 ± 4.6	26.28 ± 1.4	15.86 ± 1.4	32.97 ± 4.7	38.09 ± 1.9	44.10 ± 1.9	40.13 ± 1.4	40.58 ± 1.0	42.84 ± 1.5	37.73 ± 0.9
**2**	-	-	-	0.6 ± 0.2	11.3 ± 0.6	0.23 ± 1.7	22.55 ± 6.9	29.06 ± 1.4	39.29 ± 1.9	39.61 ± 1.2	39.23 ± 1.3	37.98 ± 1.3	39.56 ± 0.9
**5**	-	-	-	0	0	0	1.74 ± 0.9	6.45 ± 1.1	24.35 ± 6.1	23.83 ± 3.1	26.40 ± 3.7	27.22 ± 1.2	37.02 ± 1.0
**7.5**	-	-	-	0	0	0	0.01 ± 0.0.1	3.15 ± 1.7	14.65 ± 5.8	10.39 ± 5.1	11.20 ± 4.6	16.97 ± 2.1	37.57 ± 1.8
**10**	-	-	-	0	0	0	0	0.52 ± 0.2	14.74 ± 6.8	1.79 ± 0.9	0.09 ± 0.1	11.36 ± 2.0	40.44 ± 1.4
**12.5**	-	-	-	-	-	-	0	0.69 ± 0.4	12.04 ± 5.5	1.15 ± 0.7	0	4.63 ± 0.6	38.32 ± 0.9
**15**	-	-	-	-	-	-	0	0.47 ± 0.4	8.32 ± 3.5	0	0	2.57 ± 1.3	40.51 ± 1.9
**20**	-	-	-	-	-	-	-	0.08 ± 0.1	1.92 ± 0.6	0	0	0.25 ± 0.1	40.46 ± 1.2
